# The role of interleukin-22 in lung health and its therapeutic potential for COVID-19

**DOI:** 10.3389/fimmu.2022.951107

**Published:** 2022-07-27

**Authors:** Si Fang, Dianwen Ju, Yong Lin, Wei Chen

**Affiliations:** ^1^ Multiscale Research Institute of Complex Systems & Jingan District Central Hospital of Shanghai, Fudan University, Shanghai, China; ^2^ Department of Biological Medicines & Shanghai Engineering Research Center of Immunotherapeutics, Fudan University School of Pharmacy, Shanghai, China; ^3^ Department of Ophthalmology, Stanford University School of Medicine, Palo Alto, CA, United States

**Keywords:** interleukin-22, immunotherapeutic strategy, lung, COVID-19, SARS-CoV-2 infection

## Abstract

Although numerous clinical trials have been implemented, an absolutely effective treatment against coronavirus disease 2019 (COVID-19) is still elusive. Interleukin-22 (IL-22) has attracted great interest over recent years, making it one of the best-studied cytokines of the interleukin-10 (IL-10) family. Unlike most interleukins, the major impact of IL-22 is exclusively on fibroblasts and epithelial cells due to the restricted expression of receptor. Numerous studies have suggested that IL-22 plays a crucial role in anti-viral infections through significantly ameliorating the immune cell-mediated inflammatory responses, and reducing tissue injury as well as further promoting epithelial repair and regeneration. Herein, we pay special attention to the role of IL-22 in the lungs. We summarize the latest progress in our understanding of IL-22 in lung health and disease and further discuss maneuvering this cytokine as potential immunotherapeutic strategy for the effective manage of COVID-19.

## Introduction

Severe acute respiratory syndrome coronavirus-2 (SARS-CoV-2) infection has affected more than 548 million coronavirus disease 2019 (COVID-19) patients and caused more than 6.3 million deaths globally (https://coronavirus.jhu.edu, Johns Hopkins Coronavirus Resource Center) up to July 2, 2022, with these cases continuously increasing and producing several variants of concern. COVID-19 patients develop a constellation of clinical features, ranging from mild respiratory symptoms to severe acute respiratory syndromes, and even death (1, 2). Multiple evidences have illustrated that cytokine release syndrome (CRS) is the pathological hallmark of critically ill COVID-19 patients, which is characterized by a rapid and sustained systemic increase of more than 20 inflammatory chemokines and cytokines. CRS induced secondary hemophagocytic lymphohistiocytosis and acute respiratory distress syndrome (ARDS) are regarded as main causes of organ injuries that drive the deterioration of COVID-19 (3–7). Therefore, besides direct supplemental oxygen and antiviral therapy for COVID-19 patients, immunotherapeutic strategies potentially alleviate COVID-19 progression and rescue severe or critical illness. Unfortunately, a recent double-blind, randomized Phase III trial (clinicaltrials.gov; NCT04320615) testing the treatment efficacy of an anti-interleukin (IL-6) receptor antibody (tocilizumab) in COVID-19 cases failed to show a significant reduced the mortality (8, 9). Thus, further studies aimed at exploration of novel immunomodulatory therapeutic strategies to treat COVID-19 and development corresponding regimens are urgently warranted.

**Figure 1:**
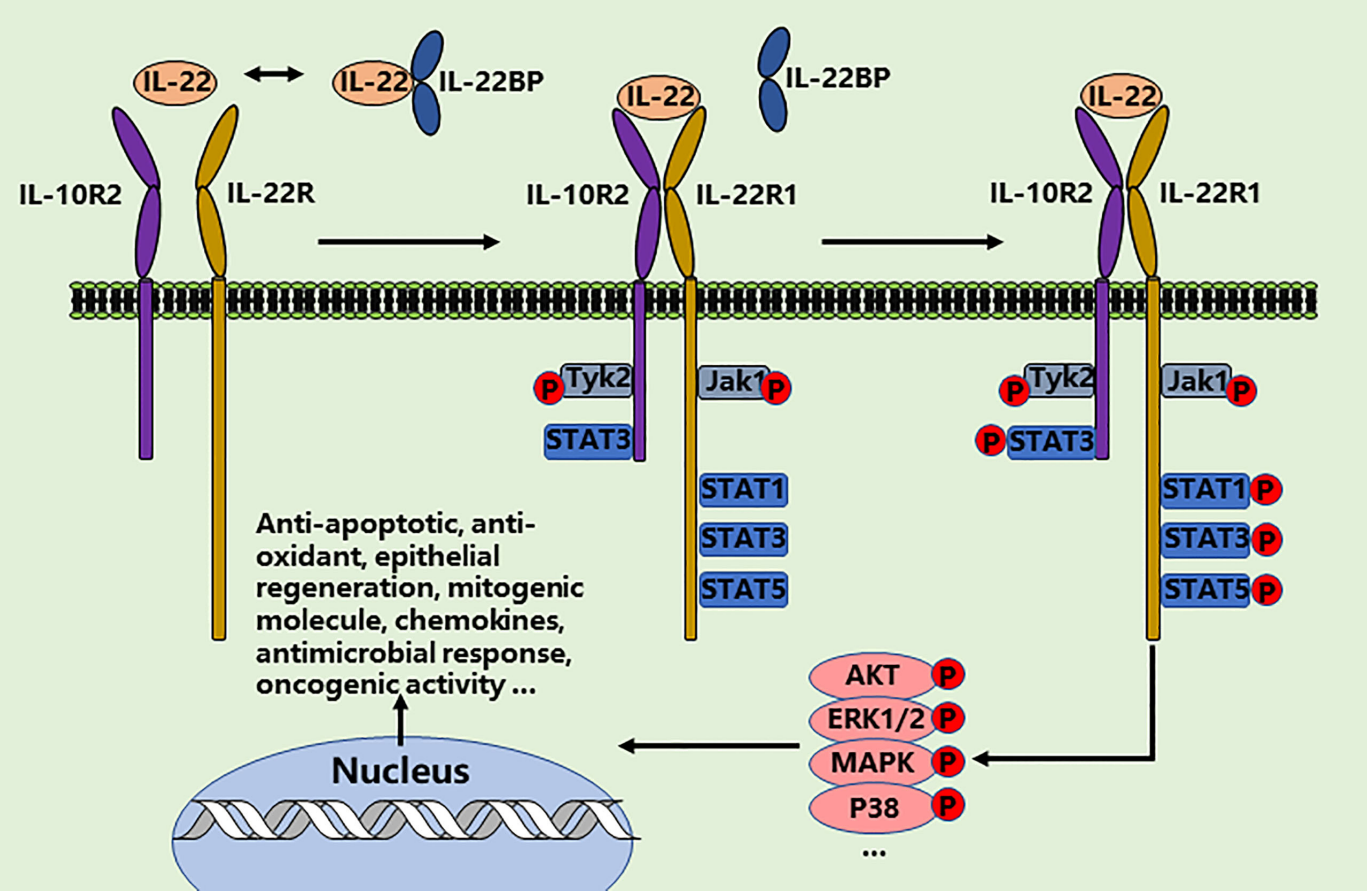
IL-22/IL-22-R1 signaling events. IL‐22 exerts the therapeutic effects in two steps through a receptor complex composed of IL‐10R2 and IL‐22R1. IL‐22-IL10R2/IL‐22R1 complex leads to the phosphorylation of JAK1 and TYK2 and then phosphorylates the tyrosine residues such as STATs, AKT, ERK1/2, MAPK, and P38 in the cytoplasmic domain of IL‐22R1. The cellular effects of IL-22 are involved in anti-apoptotic and anti-oxidant response, epithelial regeneration, mitogenic molecule expression, chemokines expression, antimicrobial response, and oncogenic activity.

Interleukin-22 (IL-22) has attracted great interest over recent years, making it one of the best-studied cytokines of the interleukin-10 (IL-10) family (10, 11). The function of IL-22 is mediated through directly interacting with its heterodimeric IL-22R1 and IL-10R2 receptor complex (12, 13). Unlike most interleukins, which directly regulate the function of hematopoietic cells, the major impact of IL-22 is exclusively on fibroblasts and epithelial cells due to the restricted expression of receptors in the kidney, lung, liver, pancreas, gastrointestinal tract, skin, and thymus (14). Therefore, IL-22 represents a main communication channel between specialized tissue cell types and the immune system. Signaling occurs *via* the activation of Jak1/Tyk2 and STAT3 pathway and then activation of anti-apoptotic, mitogenic and antioxidant molecules. Protein kinase B (AKT)/mechanistic target of rapamycin (mTOR), ERK1/2, P38, MAPK, STAT1, and STAT5 pathways are also activated by IL-22 (15–20). Of note, numerous of studies have illustrated that IL-22 plays a pivotal role in anti-viral infections through significantly ameliorating the immune cell-mediated inflammatory responses, as well as reducing lung injury and promoting further airway epithelial repair and regeneration **(** **Figure 1** **)** (21–23). Herein, we summarize recent progress in understanding the biology of IL-22 in lung health, suggesting more immunotherapeutic strategies to maneuver this cytokine for the effective manage of COVID-19 (24–28).

## Pathological characteristics of COVID-19

COVID-19 is a complex disorder in which the respiratory performance is accompanied by systemic responses, illustrating viral infection generates widely dysfunctional immune reactions. Generally, the pathological feature of COVID-19 is bilateral diffuse alveolar damage with evidence of neutrophil extracellular traps, fibrin thrombi, and activated platelets in the vessels (25, 26). Infiltrating monocytes, macrophages, and neutrophils are clearly found in both lungs, accompanied by multinucleated syncytial cells infiltration in the alveoli that characterized by amphiphilic granular cytoplasm, large nuclei, and prominent nucleoli indicating viral cytopathic alters (27–31). Additionally, myocardial infarction, kidney damage, and persistent symptoms, such as depression, anxiety, palpitations, chest pain, sleep difficulty, dizziness, and weight loss, also represent the pathological features of critically COVID-19 cases (27–32). The immune profile in COVID-19 patients has been well reviewed, which shows dysfunction in both the innate and adaptive immune (33, 34). Specifically, CD16^+^ monocytes, γδ T cells, and NK cells are obviously activated, while the percentages of CD4^+^ T cells, CD8^+^ T cells, and natural killer (NK) cells are significantly decreased (34). Besides that, T cells show hyperinflammatory responses and enhanced migration abilities, along with evidently increased expression of inhibitory molecules (7, 33, 34). The clonality of B cells and the proportion of plasma B cell compartments are also elevated. The percentages of dendritic cells are obviously decreased; however, the IFN-response cell compartments are increased (34–38). For the mechanisms, multiple evidence has demonstrated that IL-1 axis and IL-6 axis are the most importantly relevant signal transductions in the SARS-CoV-2-induced hyperinflammatory responses (39).

Altogether, the pathological characteristics of COVID-19 are complex, comprising fibrotic processes, hyperinflammation, thromboembolic complications and endothelial cells and lymphocyte dysfunction in the lung. These processes between patients are also highly variable, may be due to the heterogeneity of host immune reaction, which urgently require stratified and novel immunotherapy strategies for COVID-19 management.

## Immunotherapeutic strategies for COVID-19

Despite numerous clinical trials have been implemented, an absolutely effective treatment against COVID-19 is still elusive. Immunomodulatory medications hold huge promise, but their administration should be cautiously considered so that the protective effects are appropriate for the dominant immunopathology and the disease stages to minimize the side-effects (**Table 1**).

**Table 1:** Overview of the candidate immunotherapeutic strategies for the treatment of COVID-19/SARS-CoV-2 infection.

Drug class	Target	Trial/paper	Overall status and conclusion	Ref
**Cytokine antagonists**	Blocking IL-1 (Canakinumab, Anakinra )	NCT04362813 NCT04680949 NCT02735707 NCT04357366 NCT04341584	Canakinumab showed no efficacy in clinical trials; Anakinra had promising results and larger clinical trials are pending	(40, 41)
	Blocking IL-6 (Sarilumab, Tocilizumab)	NCT02735707 NCT04381936	Sarilumab phase 3 trials were stopped; Dexamethasone&Tocilizumab were recommended by NIH for severe cases treatment	8, 9)
	Blocking GM-CSF (mavrilimumab)	NCT04318366	Phase 2 with primary endpoint was reached	(42)
	Blocking TNF-a (Etanercept, Adalimumab),	Case series	Further studies are required	(43)
	Blocking CCR5 (Leronlimab)	NCT04678830	Currently not recommended	(44)
**Kinase inhibitors**	Blocking JAK1and JAK2 (Baricitinib)	NCT04401579 NCT04421027	Baricitinib has been approved by FDA as EUA, but the risk of adverse consequences remains	(45)
	Blocking JAK1and JAK3 (Tofacitinib)	NCT04469114	The risk of respiratory failure or death was reduced, but the risk of adverse reactions remained	(46)
**Inflammasome inhibitors**	Blocking Gasdermin D pore (Disulfiram)	Preclinical studies	Lack of efficacy and safety studies	(47)
	Blocking NLRP3 inflammasome (Melatonin)	NCT04409522	As an adjunctive therapy; further larger studies are required	(48)
**Immune stimulants**	IFNβ-1a, IFNβ-1b, IFN-𝜆	NCT04385095 NCT04276688 Case series	Nebulized IFNs has demonstrated efficacy	(49, 50)
**Immunomodulatory therapies**	Glucocorticoids	NCT02517489 NCT04244591	Only favorable results in critical cases, but accompanied by multiple adverse effects	(51)
**Neutralizing antibody**	Sotrovimab, LY-CoV555	NCT04545060 NCT04411628 NCT04427501 NCT04497987 NCT04501978 NCT04518410 NCT04634409	Have been approved by FDA as EUA, but VOCs have gradually weaken the efficacies	(52, 53)

### Cytokine immunomodulators

Of note, immunotherapeutic strategies blocking proinflammatory cytokines or their receptors are only meaningful in the hyperinflammatory stages; as in the early stages of mild lung injury or minimal inflammation, cytokines are largely desirable in fighting against virus infection. These include inhibitors of IL-1, IL-6, IL-18, TNF, CCR5 and GM-CSF or their receptors. However, some phase 2 or 3 results suggest no or little benefits in moderate and severe patients, and large-scale clinical data in COVID-19 are needed (8, 40–44, 54). Interferons (IFNs) have been used for many years to treat infectious disorders, cancers and multiple sclerosis, suggesting their potent antiviral effects for COVID-19. Although recent studies have found early nebulized IFN-α/β/𝜆 administration accelerated high-risk patients’ recovery and reduced mortality, the multinational clinical trials on hospitalized severe cases indicated subcutaneous IFNs injection with or without lopinavir treatment had minor effects on length of hospital stay and mortality (49, 50, 55, 56).

### Kinase and inflammasome inhibitors

Small molecule inhibitors targeting cytokine-mediated signaling pathway, specifically of Janus kinases (JAKs), have also been well studied for treating COVID-19. For instance, Baricitinib and Tofacitinib were approved for emergency use authorization (EUA) because the clinical symptoms improved when co-treated with remdesivir during SARS-CoV-2 infection. But secondary infection and thromboembolism are the risk of these kinase inhibitors, which is highly dependent on administration time (45, 46). Glucocorticoids, such as dexamethasone, are broad-acting and powerful immunosuppressive and antiphlogistic therapies that can reduce mortality in critical COVID-19 cases (51). However, many clinical studies demonstrated that such efficacies are inevitably accompanied by multiple adverse effects, including viral reactivation, blocking of the host immune responses important for viral clearance, and others (51). In addition, it is indisputable that inflammasomes contribute to the pathogenesis of COVID-19, and trials on the GasderminD-pore inhibitor Disulfiram and the NLRP3 inhibitors Melatonin are ongoing (47, 48).

### Neutralizing antibodies

Monoclonal antibody (mAb) is one of most effective immunotherapeutic strategies for the treatment of serious viral infection; thus, lots of mAbs are identified purposing to inhibit SARS-CoV-2 infection through binding to the spike protein. So far, the FDA has approved EUA for LY-CoV555 and Sotrovimab in pediatric and high-risk patients to prophylaxis and treat COVID-19, as these antibodies can reduce mortality and hospitalization (52, 57). Nevertheless, viral mutations present in variants/variants of concern (VOCs) have begun to gradually weaken the efficacies of many mAbs, and it is likely some will become invalid without upgrading to compensate for SARS-CoV-2 evolution (53). Altogether, immune system dysfunction plays a key role in COVID-19 pathogenesis and immunotherapeutic strategies for SARS-CoV-2 infection are promising, future efforts to discover more effective agents are obviously warranted. Also note that COVID-19 remains a field of rapid progress, thus recommendations on drugs and biomarkers will continue to evolve.

## Interleukin‐22 biology in the lung

In the lung, stimulation of CD11b^+^-DCs and alveolar macrophages *via* their innate pattern recognition receptors leads to the secretion of related proinflammatory cytokines, expression of RORγt, differentiation of ILC3, γδT, Th17 and NKT cells, and production of IL-22 (57). Conversely, CD103^+^-DCs and plasmacytoid can be stimulated through viral antigens, resulting in the secretion of IL-27, IFN-γ and type I IFNs that block IL-22 expression in lymphocytes (58).

### Molecular mechanism

**Figure 2:**
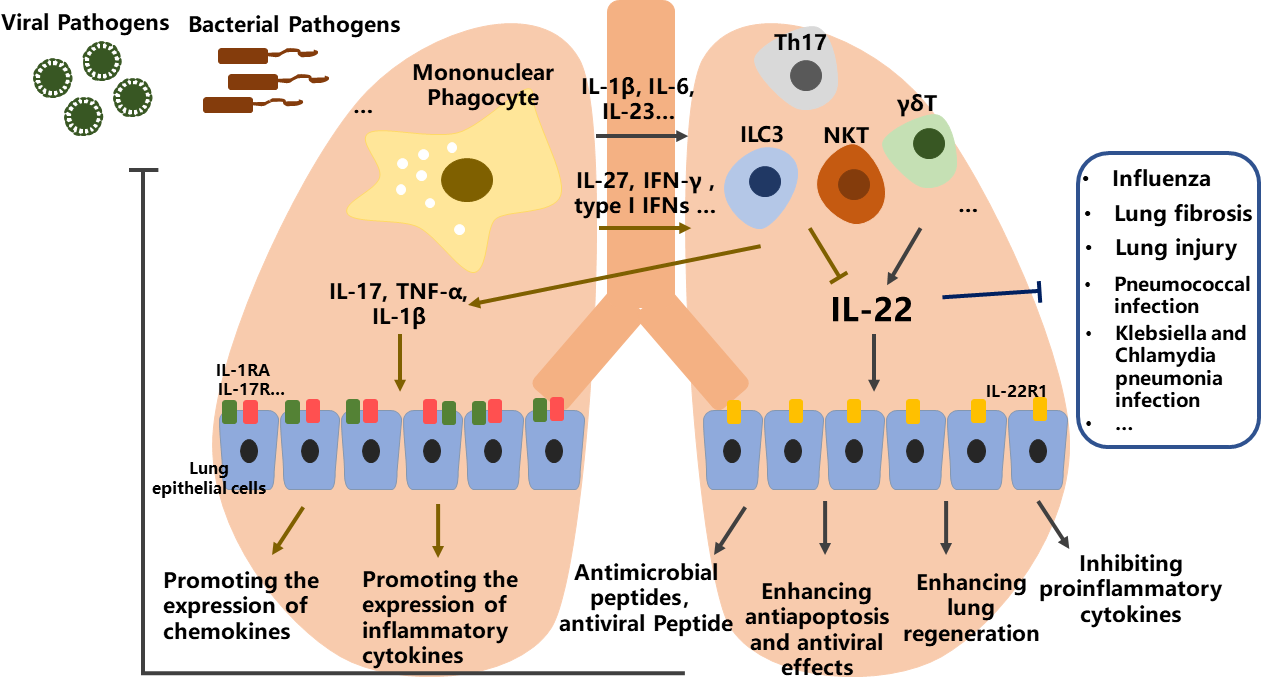
The role of IL-22 in lung health and diseases. In the lung, stimulation of mononuclear phagocyte *via* viral or bacterial pathogens leads to the secretion of related proinflammatory cytokines, then differentiation of ILC3, γδT, Th17 and NKT lymphocytes, and promoting or blocking of IL-22 expression. IL-22 can be protective or proinflammatory through multiple effects, where IL-22/IL-22R axis is important for host protective immunity to both viral infections and bacterial infections.

Multiple studies have shown that IL-22 is involved in lung repair, recovery and regeneration during lung infection or injury. From these studies, it is concluded that IL-22 can be protective or proinflammatory, where IL-22/IL-22R axis is important for host protective immunity to both viral infections and bacterial infections. The tissue protective nature of IL-22 *via* enhancing wound healing and the epithelial barrier through promoting antimicrobial peptides expression, enhancing anti-apoptosis and antiviral effects and alleviating airway inflammation, as well as inhibiting proinflammatory cytokines produced by airway epithelial cells (**Figure 2**). In contrast, the proinflammatory of IL-22 is evidenced by its ability to promote the expression of chemokines, and inflammatory cytokines such as IL-1β, IL-17, and TNF-α. Whether IL-22 has a protective or proinflammatory effect seems to depend on the related cytokines co-produced by the relevant cells during different stages of the diseases (10, 14).

### The regulation IL-22

**Figure 3:**
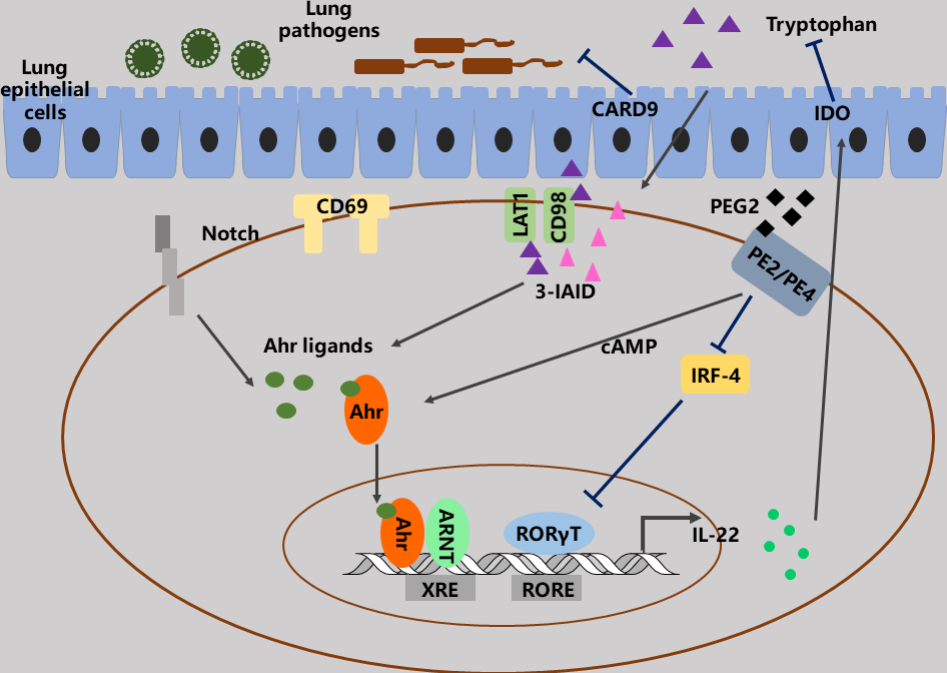
The functions and regulation of IL-22. AhR plays importance roles in the functions and regulation of IL-22 in lungs. A lot of signaling, such as PGE2, LAT1, CD69, CD98 and Notch can converge to AhR signaling to regulate IL-22 production *via* modulating of AhR ligand.

Transcription factors play crucial roles in controlling the production of IL-22 in lungs (15, 59–61). Interestingly, studies indicate that these transcription factors, such as aryl hydrocarbon receptor (AhR), c-Maf, Batf, and Notch, can sometimes control IL-22 even in the same cells. These transcription factors form complex regulatory networks and regulate the production of IL-22 in context-dependent manners (62–64). As a cytosolic transcription factor, AhR is a harbor that converges several different environmental and cellular signaling to regulate the development of many Th cell lines, including Tr1, Th17 and Treg cells *via* recognizing multiple natural molecules and small xenobiotics (62–65). AhR directly binds to cMaf and synergistically enhances IL-22 production in ILC3s, Th17 cells, γδ T cells and Th22 cells. Nevertheless, under some certain conditions, AhR may not produce IL-22, and AhR^-/-^ mice can develop severe skin inflammation associated with higher IL-22 and IL-17 expression. The Notch signaling also promotes IL-22 expression *via* AhR induction in Th17 cells (65). Additionally, prostaglandin E2 (PGE2) enhances IL-22 production from both T cells and ILC3 *via* binding EP4 and EP2 receptors and blocking IRF-4, as well as activating AhR and cAMP signaling (66, 67) (**Figure 3**). In the lungs, certain viral or bacterial microorganisms can convert tryptophan (Trp) into derivatives that promote IL-22 secretion. Of importance, IL-22 production from γδ T cells is manipulated through AhR- and Try-dependent mechanism *via* CD69 (68). And CD69 regulates LAT-1 expression, which determines the intracellular quantity of AhR and the uptake of Trp (**Figure 3**). Moreover, both CARD^-/-^ and IDO^-/-^ mice can alter downstream Trp metabolites, for instance 3-IAID, with one depriving IL-22-promoting derivatives and the other favoring them, respectively (69). Taken together, these findings illustrate that controlling AhR signaling and Trp metabolites may be a powerful tool for regulating IL-22 functions.

### Protective effects of IL-22

During the early stage of influenza infection, studies has previously illustrated that IL22 is overexpressed by NK and NKT cells in the lung, resulting in the airway and parenchymal epithelium regeneration. Within two days after viral infection, IL-22 gene transcription in Tγδ, Tαβ and innate lymphoid cells is also increased in lung (**Table 2**). However, IL22BP gene transcription is decreased. During the sublethal stage of viral infection, IL-22 can inhibit lung inflammation, reduce secondary infection and preserve the integrity of lung epithelium (70). Whereas IL-22^-/-^ mice infected with viral shows enhanced collagen accumulation and a defect in epithelial regeneration (70). Moreover, IL-22 can also prevent pulmonary fibrosis in a Bacillus subtilis induced model. Administration of IL-22 alleviates lung fibrosis, while inhibition of IL-22 leads to increased collagen accumulation in the lungs (71). In pneumococcal pneumonia, IL-22 gene is found to increase in the lung together with other cytokines, for example TNFα, IL-6, and IL-1. The absence of IL-22 makes the host vulnerable to pneumococcal infection, which indicates that the presence of IL22 is very important to control pneumococcal infection (72). IL-22 also plays a key role in host resistance to many other lung pathogens. During Klebsiella pneumonia infection, NK cells activation results in IL-22 production to protect lung tissue, and during infection with Streptococcus pneumoniae, TLR5 and DC cells activation leads to a repaid accumulation of ILC3s in the lungs to express IL-22 and defense against bacterial infection (73–75). During Chlamydia muridarum infection, IL-22 levels are rapidly increased in the lungs. Neutralization of IL-22 with anti-IL-22 mAbs can lead to impaired Th17 responses and deterioration of disease. Intranasal injection of IL-22 can significantly enhance Th17 response and have a protective effect following Chlamydia pneumoniae infection (76). After Aspergillus fumigatus pulmonary infection, the fungal burden is increased in IL-22^-/-^ mice, suggesting the critical role of IL-22 in the elimination of this pathogen (77). Additionally, IL-22 can also play a protective role in the barotrauma model, thereby reducing pulmonary edema and disintegration (78).

**Table 2:** Immunotherapeutic strategies *via* targeting IL-22, in lung diseases.

Diseases	Type	IL-22 treatment	Observed effect	Ref
**Influenza**	Viral	IL-22 deficient mice and recombinant IL-22 injection	Inhibits lung inflammation, reduce secondary infection and preserve the integrity of lung epithelium	(59)
**Bacillus** **subtilis infection**	Bacterial	Intratracheal treatment with recombinant IL-22	Protection from lung fibrosis	60)
**Pneumococcal pneumonia infection**	Bacterial	Systemically administration of recombinant IL-22	IL22 is important for controlling pneumococcal infection	(61)
**Klebsiella pneumonia infection**	Bacterial	IL-22 neutralizing antibody and recombinant IL-22 injection	Defenses against bacterial infection	(62, 64)
**Streptococcus pneumoniae infection**	Bacterial	Endogenous IL-22	Defenses against bacterial infection	(63)
**Chlamydia muridarum infection**	Chlamydia	IL-22 neutralizing antibody and intranasal injection	Significantly enhances Th17 response and has protective effects	(65)
**Aspergillus fumigatus pulmonary infection**	Fungal	IL-22 neutralizing antibody and IL-22 deficient mice	Lung defense against Aspergillus fumigatus is mediated by IL-22 production	(66)
**Ventilator-induced lung injury**	Tissue damage	Prophylactic inhalation of IL-22	Alleviation of ventilator induced lung damage	67)
**Bleomycin induced lung injury**	Tissue damage	IL-22 neutralizing antibody and recombinant IL-22 injection	Proinflammatory effect of IL-22 depends on the synergistic effect with IL-17	(68, 69)
**Asthma models and asthmatic patients**	asthmatic	IL-22 neutralizing antibody and recombinant IL-22 injection	IL-22 induces the occurrence of asthma and ameliorates inflammation during asthma exacerbation	70–74)

### Proinflammatory effect of IL-22

In the animal model of bleomycin induced lung injury, it is demonstrated that IL-22 and IL-17 are predominantly produced by Th17 cells. Inhibiting IL-22 during bleomycin injection can improve airway inflammation, indicating IL-22 has a proinflammatory effect; nevertheless, airway inflammation is also significantly reduced in IL-17^-/-^ mice with still high levels of IL-22 produced by T cells, suggesting IL-22 has a protective function for pulmonary fibrosis in deficiency of IL-17 (79, 80). Therefore, the proinflammatory effect of IL-22 may depend on the synergistic effect with IL-17 (**Table 2**).

### Dual effects of IL-22 in asthma

In peripheral blood of asthmatic patients and preclinical asthma models, serum IL-22 levels are increased. The recent studies have indicated IL-22 can induce the occurrence of asthma in preclinical models, but it can ameliorate inflammation during asthma exacerbation (81, 82). In ovalbumin induced asthma animal model, IL-22 neutralization with mAbs during sensitization stage of disease, which is similar to the pulmonary fibrosis research just mentioned above, evidently alleviated lung pathology and airway inflammation. Subcutaneous administration of IL-22 during sensitization leads to worse lung pathology and inflammation (83–85). Conversely, IL-22 neutralization after sensitization can result in increased lung inflammation, whereas IL-22 injection decreases chemokine expression, goblet cell hyperplasia, production of IL-25, eosinophil infiltration and constriction of the airway (82–85). The mechanism behind this dual effect in allergic airway inflammation is unclear, but it may involve inhibiting the production of IL-25 and CCL17 through airway epithelial repair, which may involve IL-10.

In conclusion, IL-22 is involved in a variety of lung diseases, making it a promising target for clinical development.

## Potential therapeutic effect of IL-22 for COVID-19

The emergence of SARS-CoV-2, which leads to COVID-19, is one of the most serious public health and epidemics crises in this century. As an emerging virus, there are multiple problems to be clarified in distinguishing the effective immune response of mild and severe diseases.

### IL-22 in COVID-19 patients

**Figure 4:**
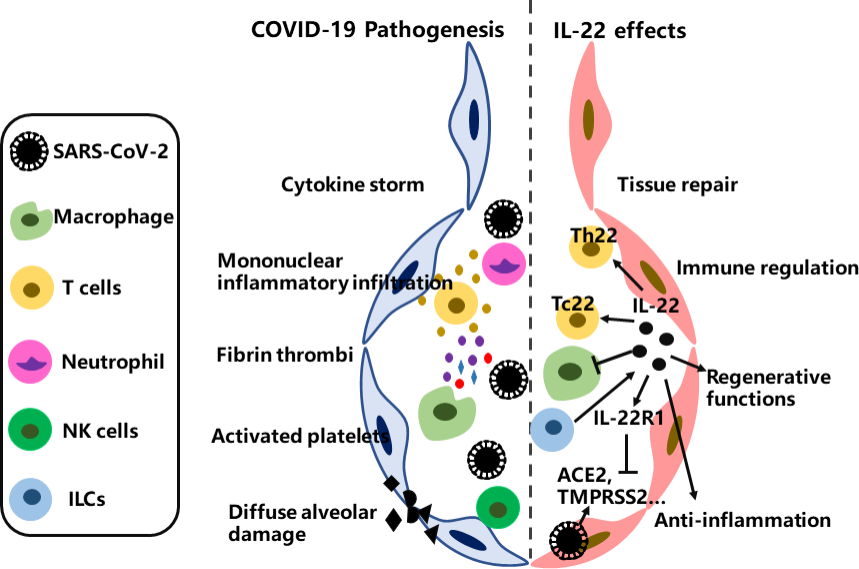
The therapeutic potential of IL-22 for COVID-19. SARS-CoV-2 virus first occupied the airway epithelial barrier; mononuclear inflammatory infiltration increased; and then platelets activation, cytokine storm, fibrin thrombi and diffuse alveolar injury followed. IL-22 can reduce the pneumonia severity through immunomodulation, anti-inflammation and tissue regenerative and repair functions, but the detailed mechanism underlying the therapeutic consequences for COVID-19 remains to be fully clarified.

Although IL-22 does not appear to reduce virus titer during infections, studies have illustrated that IL-22 can reduce the pneumonia severity *via* the immune regulation and tissue protective or regenerative functions (86, 87). As COVID-19 is a respiratory disorder with similar pathological characteristics and symptoms to other serious pulmonary virus infections, it is reasonable to speculate that IL-22 may also serve to limit the severity of this disease. More importantly, recent studies have shown that IL-22 has potent immune boosting, antiviral, and antibacterial properties to respiratory syncytial virus, which could also extend to manage SARS-CoV-2 infection (88). Accordingly, compared with healthy control individuals, the IL-22^+^-Tc22 and IL-22^+^-Th22 numbers in adult-COVID-19 patients increased significantly, whether it is asymptomatic pneumonia, mild pneumonia, or severe pneumonia **(** **Figure 4** **).** These findings further suggest that in the 0-12-year-old age group with asymptomatic disease course and uncomplicated adult cases, IL-22 expressed Tc22 cells are higher, which indicates that IL-22 has a protective effect (89). In contrast, the association between the increase of Tc17 cells and the severity of COVID-19 may reveal the destructive effect of co-expression of IL-17 and IL-22.

### IL-22/IL-22R1 axis in COVID-19 patients

As the IL-22/IL-22R1 axis is involved in inflammation during virus infection, the expression patterns of IL-22/IL-22R1 on blood hematopoietic cells in SARS-CoV-2 infection have been well studied (90–93). The numbers of IL-22R1^+^ myeloid DC1, myeloid DC2, and plasmacytoid DC and the proportions of IL-22R1^+^ intermediate, non-classical, and classical monocytes higher in COVID-19 patients than controls at the presented day. Moreover, high proportions of mDC2 and IL-22R1^+^ non-classical monocytes show high HLA-DR expression and are therefore activated. Multivariate analysis is performed on all IL-22R1^+^ myeloid cells to distinguish the disease severity. However, correlation analysis between the concentration of plasma chemokines and IL-22R1^+^ cell subsets indicates that some subsets have protective effects, while others have pro-inflammatory effects (93, 94). Without stimulation, CD4^+^-T and NK lymphocytes produced IL-22. CD4^+^-T cells expressed IL-22R1, and its expression density defines two different functional subsets. The number of IL-22R1^+^ intermediate monocytes is negatively correlated with IFN-α, CRP and IL-6 in non-serious SARS-CoV-2 infection, whereas pDC and IL-22R1^+^ classical monocytes are positively correlated with pro-inflammatory chemokines IP-10 and MCP-1 in serious SARS-CoV-2 infections. Besides, researchers further demonstrate that IL-22 can reduce the expression of viral entry receptors, such as ACE2, TMPRSS2, and increase the expression of anti-viral proteins through IL-22/IL-22R1 pathway **(** **Figure 4** **)** (95). Thus, these findings suggest that the IL-22/IL-22R1 signaling is involved in the pathological process of COVID-19, which can be protective during SARS-CoV-2 infections (93–95).

### ILCs in COVID-19 patients

Recently, studies have demonstrated the roles of ILCs (innate lymphoid cells) in COVID-19 patients. Blood from severely infected COVID-19 patients were found that had fewer ILC precursor cells and ILCs than those mild cases. Of importance, in these severely infected COVID-19 patients, the expression of CD69 in ILCs was higher, which as a marker for tissue homing and activation. ILCs-CD69^+^ increased and blood ILCs decreased in severe infections, indicating that severely infected COVID-19 patients have more lung homing and activation (96). Further findings corroborate these results by confirming that higher ILCs abundance in blood was associated with shorter hospitalization. Additionally, the number of ILCs in the blood of hospitalized patients with SARS-CoV-2 infection decreased by 1.8 times (97). Collectively, these studies demonstrate that there are correlations between severe COVID-19 and decreased ILCs in the blood. As IL-22 plays critical functions in epithelial barrier integrity and ILCs are identified as the major source of IL-22 in response to lung pathogens stimulation (98), further studies are urgently needed to assess their exact roles in SARS-CoV-2 infection since current results regarding IL-22 and ILCs in COVID-19 individuals are obtained *via* analysis of blood periphery (**Figure 4**).

## Concerns of Interleukin‐22 application

Although IL-22 possesses definite immunomodulatory properties and tissue-protective effects, mainly *via* inhibiting apoptosis and promoting proliferation of epithelial cells, these same effects have also been involved in pathological states such as psoriasis, rheumatoid arthritis, and malignant tumors (99, 100). Additionally, functions of IL-22 have been implicated in host defense within barrier-tissues such as the skin, oral mucosa, and intestine (100–102). The functional outcomes of IL-22 in immunomodulation may be either protective or pathologic, suggesting that it is an extremely controversial interleukin (96–98). Systemic administration of IL-22 can upregulate the expression of proinflammatory cytokines including G-CSF and IL-6, and chemokines like chemokine (C-X-C motif) ligands CXCL1, CXCL5, CXCL9, which is sufficient to cause an inflammatory response (99). Studies indicate that IL-22 serum levels in psoriasis and rheumatoid arthritis patients are much higher than that the health individuals, and these also correlate with the disease severity (103, 104). Of note, IL-22^-/-^ mice significantly alleviate collagen induced arthritis (105). In rheumatoid arthritis tissues, synovial fibroblasts are considered to be the key cellular target of IL-22, which may drive the proliferation of this cell type through STAT3 (106). Besides, synovial-fibroblasts activation *via* IL-22 can upregulate the expression of NF-KB ligand and CCL2, which promote inflammation and joint destruction (106, 107). Inhibition of IL-22 biological activity can also reduce the severity of psoriasis, which is also consistent with the psoriasis like symptoms in IL-22 transgenic mice (108–110). Keratinocytes are targets of IL-22 in psoriasis, and IL-22 regulates the expression of key inflammatory parameters by keratinocytes, including matrix metalloproteinases-1, IL-20 and CXCL5 (108–111). It is worth mentioning that studies have also suggested that IL-22 may also promote lung pathology during chronic exposure to Aspergillus fumigatus (112). As overactivation of STAT3 in a variety of human tumors, IL-22 is considered to be associated with many tumorigeneses (113, 114). Similarly, elevated levels of IL-22 can also be detected in human cancers, including hepatocellular carcinoma and gastric cancer as well as non-small cell lung cancer (15). The related consequences of high levels of IL-22 have been fully illustrated in the diethylnitrosamin induced hepatocellular carcinoma model, where IL-22 transgenic mice show increased and IL–22^-/-^ mice decreased tumor formation (115). These complicated biological functions result in the difficulties of IL-22 clinical application.

## Conclusions and future perspectives

In summary, it can be concluded that IL-22 is a crucial modulator of epithelial homeostasis and a regulator of host defense in the lung. It provides communication channels that allow hematopoietic cells, especially lymphocytes, to trigger pleiotropic responses in the epithelium to maintain barrier homeostasis against pulmonary pathogens while protecting the lungs from invasion or damage. IL-22 participates in various lung diseases through epithelial protection or regeneration, making it an extremely attractive cytokine for the treatment of COVID-19. Of note, the FDA has approved several research groups to study the efficacy of IL-22 during SARS-CoV-2 infection (116–118). These clinical trials suggest that IL-22 treatment can shorten the duration of Intensive Care Unit (ICU) stay. Therefore, it is necessary to further comprehensively understand the specific mechanisms and functions of IL-22 in regulating the lung microenvironment, which could enable to identify novel immunotherapeutic strategy for COVID-19. However, IL-22 has been demonstrated to promote tumor growth and accelerate inflammation in a few animal models. The therapeutic consequences of IL-22 in either direction for COVID-19 treatment should be evaluated, which will provide useful insights into its role in lung health.

## Author contributions

WC and SF wrote the manuscript. WC, YL and DJ designed the structures and supervised the work. The final version of this paper has been approved by all authors.

## Funding

Our work is partially supported by Shanghai Municipal Science and Technology Major Project [No.2018SHZDZX01] and National Natural Science Foundation of China.

## Conflict of interest

The authors declare that the research was conducted in the absence of any commercial or financial relationships that could be construed as a potential conflict of interest.

## Publisher’s note

All claims expressed in this article are solely those of the authors and do not necessarily represent those of their affiliated organizations, or those of the publisher, the editors and the reviewers. Any product that may be evaluated in this article, or claim that may be made by its manufacturer, is not guaranteed or endorsed by the publisher.
